# Sex differences in inflammatory cytokine production in hepatic ischemia-reperfusion

**DOI:** 10.1186/1476-9255-3-16

**Published:** 2006-12-19

**Authors:** Elahé T Crockett, William Spielman, Shadi Dowlatshahi, Jun He

**Affiliations:** 1 Departments of Physiology & Division of Human Pathology-College of Human Medicine, Michigan State University, East Lansing, USA

## Abstract

**Background:**

The inflammatory response to hepatic ischemia-reperfusion (I/R) is associated with an increase in cytokine production. Studies have documented that sex hormones modulate both the innate and adaptive immune responses, and that females are more robust than males. The aim of this study was to determine whether a sex difference in cytokine response to hepatic I/R exists under normal pathophysiologic condition without hormone intervention.

**Methods:**

Adult C57BL/6 mice underwent 90 min of hepatic ischemia followed by various reperfusion periods (0, 1.5, 3, 6 hr). Plasma cytokine TNF-α, IL-6, MIP-2, and KC were measured. Liver injury was assessed by plasma alanine transaminase (ALT) levels and liver histopathology.

**Results:**

A reperfusion time-dependent increase in hepatocellular injury was observed in both males and females, as indicated by increasing levels of plasma ALT and liver histopathology. The plasma cytokines were significantly increased in both female and male I/R groups compared to their respective sham counterparts. However, there was a significant difference in cytokine kinetics between the female and male I/R groups. Female mice initially had a higher level of IL-6, KC, and MIP-2 in response to I/R, which began to decline after 3 hr of reperfusion and were significantly lower than the male I/R counterparts by 6 hr of reperfusion. In contrast, the hepatocellular injury and TNF production were only moderately lower in female IR than male IR.

**Conclusion:**

The study underscores role of the gender in differential inflammatory cytokine expression in response to hepatic I/R, which may reflect the host response outcome.

## Background

Hepatic ischemia/reperfusion (I/R) injury occurs when there is a disruption of the blood flow to the liver followed by restoration of blood flow. This injury usually manifests itself during hepatic surgery, transplantation, trauma and hemorrhagic shock, and can lead to liver injury. Hepatic I/R injury is exemplified by a biphasic pattern – the early phase is characterized by Kupffer cell activation, release of pro-inflammatory cytokines, and the generation of reactive oxygen species (ROS), while the late phase is characterized by a massive neutrophil infiltration and further production of the inflammatory mediators [1].

Although it is known that cytokines, neutrophils and ROS play a role in mediating the inflammatory response during I/R injury, the exact mechanism has yet to be elucidated. It is believed that Kupffer cells, which are the largest fixed population of macrophages in the body, play an important role in initiating a pro-inflammatory response by way of cytokine and ROS production.

There is increasing evidence that a sex difference exists in regard to inflammatory response and that this difference may be attributed to the sex steroids. Although the exact mechanism by which these steroids act in regard to inflammation is unknown, it has been suggested that testosterone has immunosuppressive effects while estrogen has immunoprotective effects [2]. Specifically, estrogen has been shown to have anti-inflammatory properties, strong anti-oxidative actions, and can stimulate the anti-oxidant defense system thereby protecting tissues from oxidative damage [3]. Studies have shown that not only do females with sepsis have a lower mortality than male patients with sepsis, but that females also have a better prognosis. In particular, it has been found that male gender provides a risk factor for sepsis [4] and that treatment with estrogen has a protective effect [5]. Furthermore, it has been demonstrated that women who developed sepsis following surgical treatment had a higher level of IL-10 and a lower level of TNF-α compared to men [6]. Moreover, there has been increasing evidence indicating that menopause is associated with an increase in proinflammatory cytokines such as IL-1, IL-6 and TNF-α [7,8] and that treatment with estrogen has been shown to inhibit these cytokines [9].

Gender dimorphism has also shown a role in hepatic injury [10]. In a study by Harada *et al*. it was found that female mice had a better survival rate than their male counterparts in a model of hepatic I/R with liver reduction (i.e. hepatectomy) [11]. Additionally, male mice treated with estradiol not only showed an improved survival rate after hepatic I/R, but also showed a decrease in liver injury compared to their male control counterparts. Inaba *et al*. showed that ovariectomized female rats subjected to hepatectomy followed by administration of lipopolysaccharide had a reduced survival rate compared to their matched female counterparts, further supporting the notion that sex steroids may play a role in hepatic injury [12].

Studies noted above underscore the role of gender in experimental inflammatory tissue injury by applying variations of sex hormone interventions (i.e., administration of sex hormone, ovariectomy, etc.), in which one has control over the sex hormone levels in the experimental settings. The current study was undertaken to examine the role of gender dimorphism in hepatic I/R inflammatory response under normal pathophysiologic condition without any artificial hormone intervention. In particular, the aim was to determine whether gender difference played a role in cytokine regulation in hepatic IR. The results indicated a significant difference in cytokine regulation in response to hepatic I/R between males and females, and that the cytokine network is not the sole modulator of the hepatocellular injury during early phase of hepatic I/R injury.

## Methods

All chemicals were purchased from the Sigma Chemical Co. (St. Louis, MO), unless otherwise noted.

### Experimental animal model of hepatic ischemia and reperfusion

All animals received humane care in compliance with the Guide for the Care and Use of Laboratory Animals (National Institutes of Health Publication No. 85–23, revised 1985). Experimental protocols were reviewed and approved by the Michigan State University Animal Use and Care Committee. Adult (8–10 wk) male and female C57BL/6 mice (Charles River Laboratories, Portage, MI) weighing between 21–27 g were fed a standard diet and acclimated in the animal housing area for 1 week before experimentation. The mice were randomly divided into three groups – the test group, the sham group, and the control group. The test group underwent I/R, while the sham group underwent the same surgical protocol but without vascular occlusion to induce IR. No surgical procedure was performed on the control group. A model of partial hepatic I/R as previously described by our laboratory was applied [13,14]. This model induces severe ischemic insult to the liver without inducing hypertension and subsequent bacterial translocation into the portal venous blood. The mice were lightly anesthetized with inhaled methoxyflurane (Baxter Caribe, Inc., Guayama, PR), followed by an *i.p*. injection of 35 mg/kg sodium pentobarbital (Abbott Laboratories, North Chicago, IL). A midline laparotomy was performed, the portal circulation to the median and left lateral lobes of the liver was carefully dissected, and an atraumatic vascular clip (Accurate Surgical and Scientific Instruments Corp. Westbury, NY) was placed on the vessels, interrupting the portal venous and hepatic arterial blood supply to these lobes. The abdomen was temporarily closed with sterile staple sutures to prevent dehydration and possible contamination. After 90 min of partial hepatic ischemia, the clamp was removed and reperfusion resumed. The abdomen was closed in a double layer using 5-O nylon, and 0.8 ml sterile lactated Ringer's Solution (Abbott Laboratories, North Chicago, IL) was administered subcutaneously to compensate for operative fluid loss. During the reperfusion, the mice were kept in clean cages with no further administration of anesthesia or analgesics. After reperfusion, mice were euthanized, and blood and tissue samples were collected, as described below.

All the surgical procedures were performed under aseptic conditions. In this model, the caudate and right lateral lobes, as well as the papillary and quadrate processes, retained an intact portal and arterial inflow and venous outflow to prevent intestinal venous congestion. This resulted in the induction of ischemia to approximately 70% of the liver. This hepatic IR model with 90 min of ischemia induces a severe, isolated, and reproducible liver injury with very minimal animal mortality within 2 weeks of the injury (i.e. 0–5%).

### Peripheral blood and tissue procurement

Blood samples were obtained from the right ventricle via a left anterior thoracotomy at the time of euthanasia. The blood was collected in a sterile syringe containing 50 μl of heparin (100 USP Units/ml), and centrifuged to separate the plasma. The plasma samples were stored at -70°C until use for cytokine and ALT assays. A portion of the ischemic and non-ischemic liver lobes were fixed in buffered 10% formalin, embedded in paraffin, and used for hematoxylin and eosin (H&E) staining. Another portion of ischemic and non-ischemic liver lobes was frozen in optimal cutting temperature compound (OTC media) and stored at -70°C until use for cryosection preparation and immunohistochemistry staining.

### Measurement of plasma alanine aminotransferase levels

The plasma ALT levels were determined spectraphotometrically, as previously described [13]. The ALT values are expressed in international units per liter (IU/L).

### Histopathology

H&E staining was performed on tissue sections prepared at 5-μm intervals from paraffin-embedded liver tissue. A pathologist, blinded to the experimental conditions, examined the liver tissue sections.

### Plasma cytokine concentrations

Plasma TNF-α, IL-6 and MIP-2 were determined in a 96-well Nunc-Immuno microplate (VWR Scientific, Chicago, IL), using a sandwich enzyme-linked immunosorbent assay (ELISA) technique, as previously described [13]. The capture antibody was a polyclonal anti-mouse TNF-α, IL-6, KC, or MIP-2 specific goat IgG (R&D Systems, Minneapolis, MN) and the detection antibody was a biotinylated polyclonal anti-mouse TNF-α, IL-6, KC or MIP-2 specific goat IgG, (R&D Systems). All plasma samples were tested in duplicate. The minimal detectable protein concentration was 20 pg/ml.

### Determination of hepatic neutrophil accumulation by immunohistochemistry

To confirm the presence of neutrophils in the liver tissue, immunohistochemical staining was performed using 5-μm thick liver tissue section. The primary antibody (clone 7/4, IgG2a) specific to mouse neutrophils (Cedarlane, Westbury, NY), the biotin-conjugated secondary antibody (PharMingen, San Diego, CA), and Vectastain avidin-biotin complex reagent and 3,3'-diaminobenzidine chrsomogen kits (Vector Laboratories, Inc., Burlingame, CA) were used as previously described in detail [14]. A matched antibody isotype immunoglobulin (IgG) was applied as a negative control antibody to monitor the anti-neutrophil antibody specificity. Tissue sections were counter stained with hematoxylin (Gill's formula, Vector Laboratories) and mounted with DAKO Mounting Media (DAKO Corp, Carpinteria, CA). The samples were examined using a Nikon light microscope interfaced with a spot 24-Bit Digital Color Camera.

### Statistical analysis

The data is expressed as means ± standard error of the mean (SEM). Comparisons between two groups were performed using an unpaired *t*-test. Comparisons between multiple groups and various time points were analyzed using ANOVA followed by a Fisher's PLSD post-hoc test. *P *≤ 0.05 was considered statistically significant. All data was analyzed using the *StatView version 5.0.1 software ^*© *^*for Windows.

## Results

### Demonstration of hepatic injury by determination of ALT levels and histopathology

**Figure 1:**
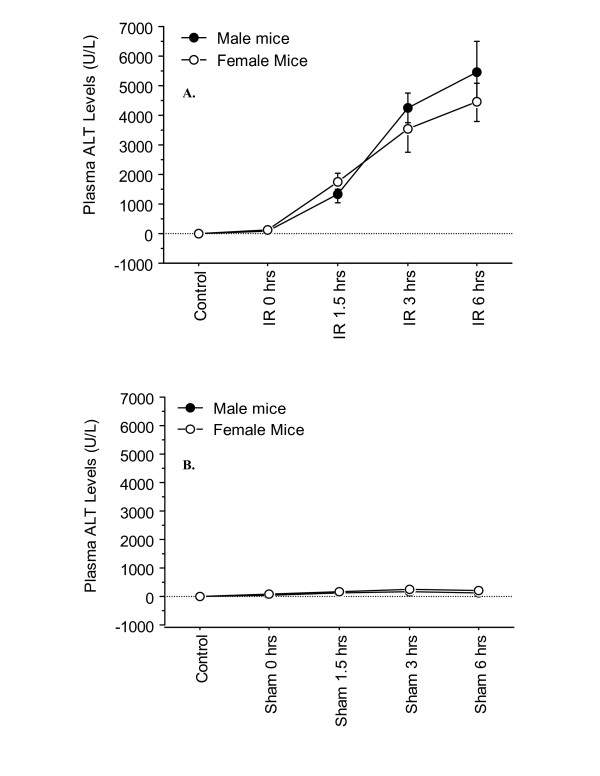
**Time-course of plasma ALT levels following hepatic I/R**. Mice were subjected to 90 min. of ischemia followed by reperfusion with various lengths of time. "Control" indicates mice that underwent no surgical procedure. "Sham" indicates mice that underwent surgical procedure with no vascular occlusion followed by reperfusion, while "I/R" denote mice that underwent I/R surgical procedure. Values are expressed as mean ± SEM. *P < 0.05 female group vs. male group (n = 6–9 mice per each time point/group). **A**: I/R group; **B**: Sham group.

**Figure 2:**
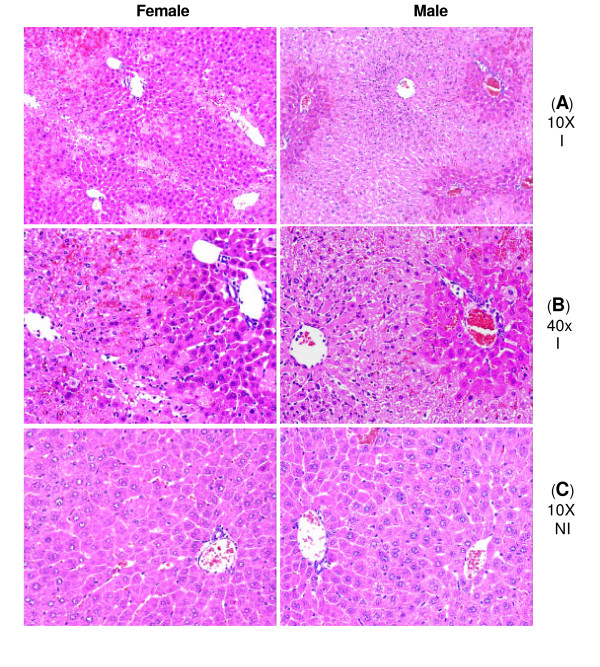
**Liver histopathology following hepatic I/R**. Mice were subjected to surgical procedure involving 90 min of ischemia followed by 6 hrs of reperfusion, and the liver sections were then prepared and stained with H&E. Images were obtained with a 10× and 40× objective lenses. Rows A and B correspond to the ischemic liver sections, while row C corresponds to the non-ischemic liver of the same animal subjected to hepatic I/R. There is a significant amount of ischemic damage, as is evident by the loss of hepatocytes in the pericentral and midzonal areas, with relative sparing of the periportal areas (Rows A and B). Neutrophils are present in the midzonal region around the central vein. The non-ischemic liver sections show normal histology (Row C). Images are representative of 6–9 liver samples per each time point/group. Abbreviations: I = ischemic liver, NI = non-ischemic liver.

Ninety min. of hepatic ischemia followed by reperfusion caused hepatocellular injury in a time-dependent fashion, as demonstrated by plasma ALT levels (Figure 1A). Significant hepatocellular injury occurred at 1.5, 3, and, 6 hrs of reperfusion in both male and female I/R mice compared to their respective sham equivalents (Figure 1A). There was no significant increase in hepatocellular injury at the onset of reperfusion (i.e. at time 0 of reperfusion), indicating that reperfusion played a critical role in the liver injury. This level of injury at 0 hrs of reperfusion was similar to those observed in sham and non-operated control groups (Figure 1A and 1B). The female I/R mice at 3 and 6 hrs of reperfusion consistently showed lower levels of plasma ALT than their respective male counterparts, although this difference did not reach a statistical significance (Figure 1A, *p *= 0.4). Further, histopathology examination confirmed hepatic injury, as was evident by sinusoidal congestion, cytoplasmic vacuolization, hepatocellular necrosis, and neutrophil infiltration. After 1.5, 3, and, 6 hrs of reperfusion, heaptocellular injury was present and the injury was maximal by 6 hrs of reperfusion (Figure 2A, and 2B). There was sparing of the periportal areas with progressive increased injury observed approaching the central vein. There was no evidence of hepatic injury due to ischemia alone, and the tissue histopathology was similar to that of sham and nonoperated control groups (Figure 2C)

**Figure 3:**
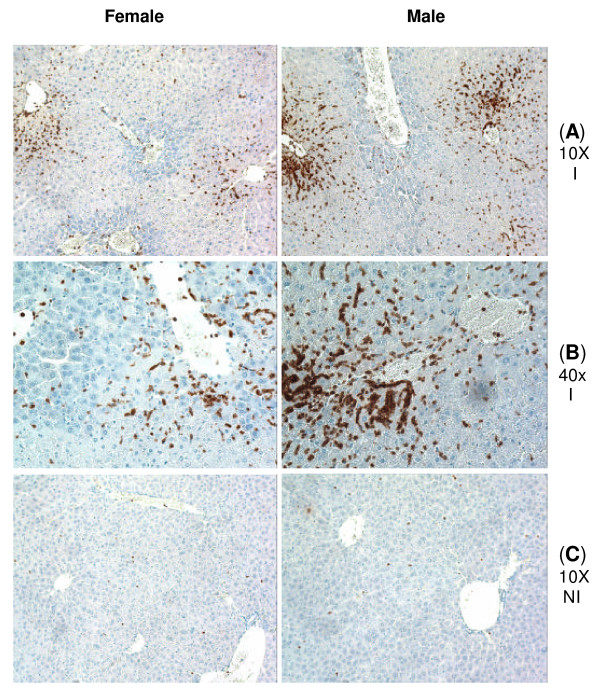
**Immunohistochemical staining of neutrophils in the liver tissue section**. Mice were subjected to surgical procedure involving 90 min of ischemia followed by 6 hrs of reperfusion. Neutrophils are stained dark brown. Rows A and B correspond to the ischemic liver sections, while row C corresponds to the non-ischemic liver of the same animal subjected to hepatic I/R. Abbreviations: I = ischemic liver, NI = non-ischemic liver. Images are representative of four liver samples per each time point/group.

An immunohistochemical technique was applied to confirm the infiltrated neutrophils in the liver tissue by using a specific antibody to mouse neutrophil. A marked increase in neutrophil infiltration was not observed until after 90 min. of ischemia followed by 6 hrs of reperfusion (Figure 3A, and 3B). Although neutrophil trafficking into the ischemic liver appeared to be more intense in male mice than the female counterparts, the overall analysis did not indicate a significant difference between the two groups (sample size of eight mice per each group). There was a minimal neutrophil infiltration into the non-ischemic lobe of the IR animals indicating that the local ischemic insult and not that of circulating cytokines/chemokines played a major role in neutrophil trafficking (Figure 3C). Neutrophil infiltration into the ischemic liver at early time-points of reperfusion (i.e., 0, 1.5 and 3 hr) was minimal (images not shown).

### Plasma TNF-α, IL-6, MIP-2, and KC Levels

Inflammatory cytokines such as TNF-α and IL-6 have shown to play key roles in pathophysiology of hepatic IR injury [15,16]. We have previously shown that in hepatic I/R, the plasma cytokine levels increase following reperfusion, reaching maximal levels between 3 and, 6 hrs of reperfusion, and that these levels return to baseline after 6 hrs of reperfusion [14]. As such, in this study the cytokine profile was examined during the first 6 hrs of reperfusion.

**Figure 4:**
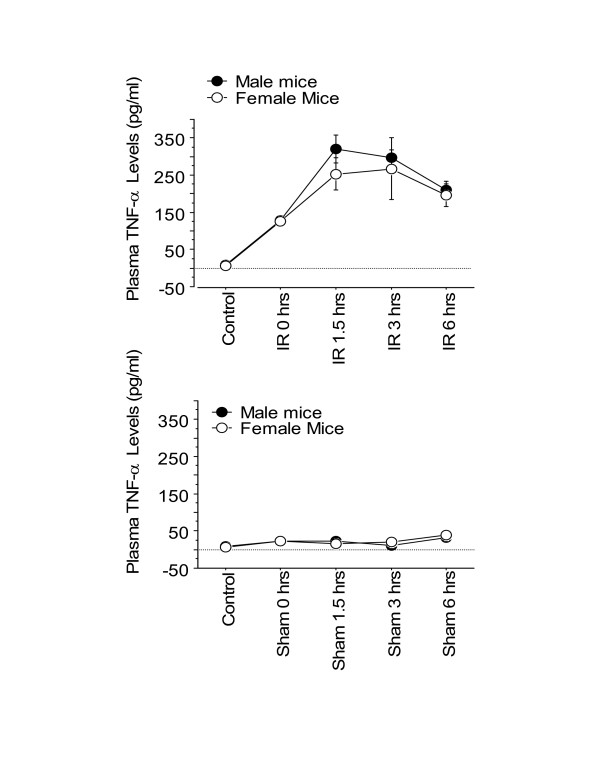
**Time-course of plasma TNF-α levels following various reperfusion times, after the onset of 90 min of ischemia**. "Control" indicates mice that underwent no surgical procedure. "Sham" indicates mice that underwent surgical procedure with no vascular occlusion followed by reperfusion, while "I/R" indicates mice that underwent surgical procedure with vascular occlusion for 90 min followed by reperfusion for various lengths of time. Values are expressed as mean ± SEM. *P < 0.05 female group vs. male group (n = 5–9 mice per each time point/group). **A**: I/R group; **B**: Sham group.

In our previous study, we demonstrated that in this model of hepatic I/R Kupffer cells are the major cellular source of cytokine production and that plasma cytokines correlated with liver mRNA of the related cytokines [14]. As such, in the current study, the plasma cytokine/chemokine levels were measured using an ELISA. There was a significant increase in plasma TNF-α level as reperfusion time increased in both male and female I/R mice compared to the sham-treated mice (Figure 4B). Minimal levels of TNF-α were observed in the sham-treated mice (Figure 4B). Although, no statistically significant difference was observed between plasma TNF-α levels of male and female I/R mice (*p *= 0.4), female TNF-α levels were lower than those of their male counterparts at 3 and, 6 hrs of reperfusion (Figure 4A). This data corroborates the plasma ALT levels, which showed a similar finding.

**Figure 5:**
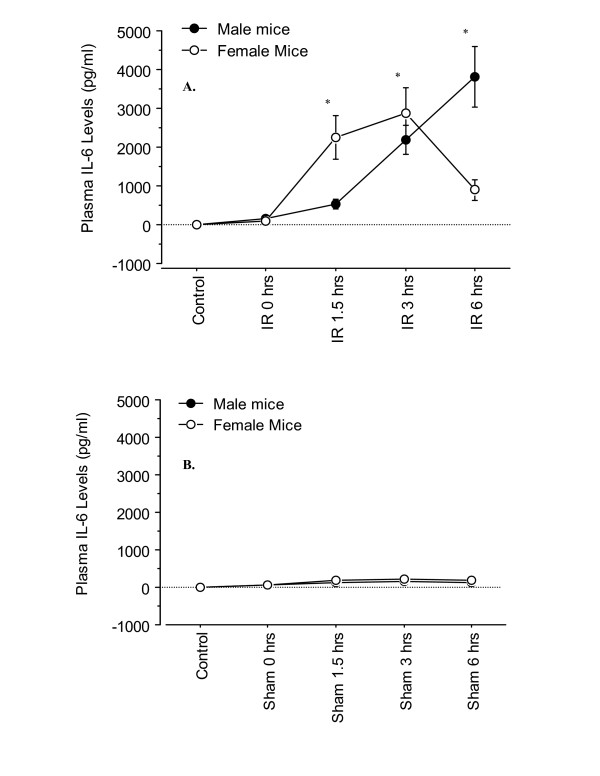
**Time-course of plasma IL-6 levels following various reperfusion times, after the onset of 90 min of ischemia**. "Control" indicates mice that underwent no surgical procedure. "Sham" indicates mice that underwent surgical procedure with no vascular occlusion followed by reperfusion, while "I/R" indicates mice that underwent surgical procedure with vascular occlusion for 90 min followed by reperfusion for various lengths of time. Values are expressed as mean ± SEM. *P < 0.05 female group vs. male group (n = 5–9 mice per each time point/group). **A**: I/R group; **B**: Sham group.

The plasma IL-6 levels were significantly increased in the I/R group compared to the sham group (Figure 5). This difference was also extended into differences between the two sexes (Figure 5A). There was a significant increase in plasma IL-6 levels at 1.5 hrs of reperfusion in the female I/R group compared to the male group. However, this difference decreased after 3 hrs of reperfusion, and by 6 hrs of reperfusion, the plasma IL-6 levels in female I/R mice were significantly lower than their respective male counterparts (Figure 5A). The plasma IL-6 levels in sham mice were minimal in both male and female group, and no difference was present between the two sexes (Figure 5B).

**Figure 6:**
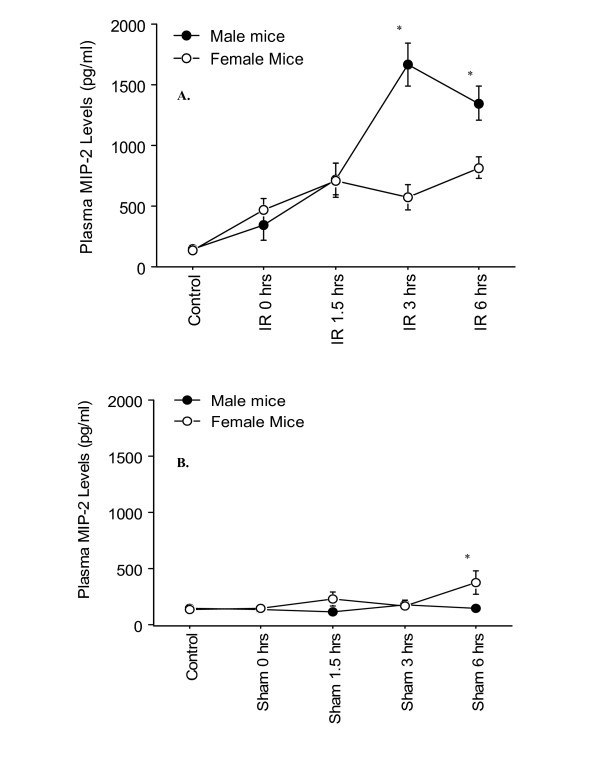
**Time-course of plasma MIP-2 levels following various reperfusion times, after the onset of 90 min of ischemia**. "Control" indicates mice that underwent no surgical procedure. "Sham" indicates mice that underwent surgical procedure with no vascular occlusion followed by reperfusion, while "I/R" indicates mice that underwent surgical procedure with vascular occlusion for 90 min followed by reperfusion for various lengths of time. Values are expressed as mean ± SEM. *P < 0.05 female group vs. male group (n = 5–9 mice per each time point/group. **A**: I/R group; **B**: Sham group.

The C-X-C chemokines, KC, and MIP-2 are potent neutrophil chemoattractants, which are important in hepatic IR injury [14,16]. In the current study, similar to the above noted cytokines, a significant increase in MIP-2 levels was observed in the I/R treated group compared to the sham group (Figure 6). In particular, there was an increase in MIP-2 levels at all time-points excluding the zero time-point. A significant difference between male and female I/R mice was observed at 3 and, 6 hrs of reperfusion (Figure 6A). Female I/R mice showed significantly lower levels of MIP-2 compared to the male mice. Although, the plasma MIP-2 levels in both sham and female sham treated groups were minimal at all time-points, the female mice at 6 hrs of reperfusion had a significantly higher level of MIP-2 than the male counterparts (Figure 6B).

**Figure 7:**
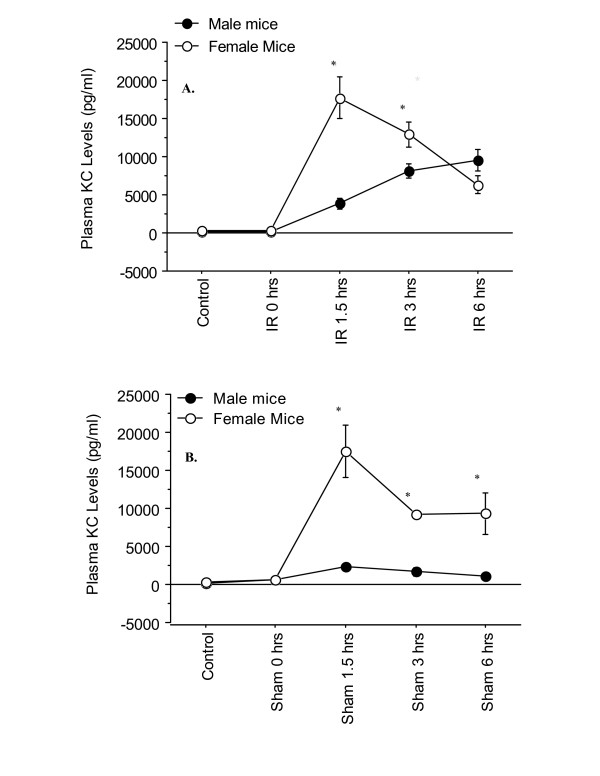
**Time-course of plasma KC levels following various reperfusion times, after the onset of 90 min of ischemia**. "Control" indicates mice that underwent no surgical procedure. "Sham" indicates mice that underwent surgical procedure with no vascular occlusion followed by reperfusion, while "I/R" indicates mice that underwent surgical procedure with vascular occlusion for 90 min followed by reperfusion for various lengths of time. Values are expressed as mean ± SEM. *P < 0.05 female group vs. male group (n = 5–9 mice per each time point/group. **A**: I/R group; **B**: Sham group.

In the current study, the plasma KC levels followed a similar pattern of elevation like IL-6 (Figure 7). In particular, it was observed that female I/R mice had higher levels of KC than the male at 1.5 and, 3 hrs of reperfusion, which declined by 6 hrs of reperfusion (Figure 7A). Of particular interest was that the female sham mice had significantly higher level of Plasma KC than their male counterparts (Figure 7B).

## Discussion

The importance of sex hormones in modulating both the innate and adaptive immune responses has been documented, which is mainly based on experimental studies in which sex steroids/drugs were administered to ovariectomized/castrated animals or tissue cultures. The role of gender differences in inflammatory cytokine regulation in response to hepatic I/R has never been examined under normal pathophysiologic condition. As such, the aim of this study was to examine the role of gender dimorphism in inflammatory tissue cytokine response to hepatic IR under normal pathophysiologic condition without any artificial sex-steroid interventions. The results of this study indicated that a statistically significant difference in cytokine/chemokine production was present between the two genders, the cytokines were differentially regulated in response to hepatic I/R, and that the hepatocellular injury was independent of the chemokine production.

Plasma ALT levels are indicative of hepatic injury. The data from the current study showed a reperfusion time-dependent liver injury increase in both sexes. However, when the responses of the two sexes were compared to one another, a small to moderate difference (i.e., 17–20%) in hepatic damage was observed, in that the males had higher levels of ALT than the females. The difference did not reach a statistical significance (*p *= 0.4). This is in contrast to previously reported data by Harada *et al*., who showed a significant difference in ALT levels, with males having higher levels of ALT [11,17]. This difference in results between these two studies may be explained by the length of reperfusion time and the animal models applied. In the studies by Harada *et al*., plasma ALT levels were measured after 20 hrs of reperfusion, unlike in the current study in which ALT levels were measured at 0, 1.5, 3, and, 6 hrs of reperfusion. The time-points in the current study were chosen as the objective was to study the cytokine/chemokine regulation and its relation to the hepatic injury. Previously published studies from our laboratory have shown that cytokine/chemokine levels during these time-points are maximal and as time progresses the levels start to decrease, reaching their baseline by 15 hrs of reperfusion [14]. The longer reperfusion time used in the Harada *et al*. studies may have allowed for greater hepatic damage to occur between males and females during the late phase of I/R, allowing for a statistical difference to be noticed. In support of this argument, a recent study from our laboratory reported a cytokine-independent hepatic injury during the late phase of liver I/R injury [13]. In addition, other factors may have influenced the difference observed, including the estrous cycle and the animal model used. The animal model used by Harada *et al*. was hepatic I/R with liver reduction (i.e., hepatectomy) whereas our model was a partial hepatic I/R model without hepatectomy [11,17]. A previous study has shown that hepatectomy induces a marked elevation of circulating sex steroids, and that estrogen has a potential role in activating gene expression, which promotes hepatic regeneration [18,19], thus allowing for a more prominent sex difference to be observed, as was seen in the Harada studies [11,17]. Further, in the current study, female mice had different levels of estrogen since they were at various stages of the estrus cycle, as determined by cyto-analysis of the vaginal preps (data not shown). This is in contrast to the other studies in which optimal concentrations of sex steroids were administered to ovariectomized/castrated animals.

Inflammatory cytokines such as TNF-α and IL-6 have shown to play key roles in pathophysiology of hepatic I/R injury [14-16]. Tumor necrosis factor-α is the proximal cytokine that is expressed following hepatic I/R, and correlates with hepatic reperfusion injury. The data from this study corroborates this, as it was similar to plasma ALT levels, TNF-α levels increased with increasing reperfusion time (Figure 4). When the TNF-α production in response to hepatic I/R was compared between the male and female mice, a small to moderate difference (*p *= 0.3 to 0.7) was noticed at 1.5, 3 and, 6 hrs of reperfusion, in that the males had higher levels of plasma TNF-α than their female counterparts (i.e., 8–20%). This observation was corroborated with the plasma ALT results. Conflicting data regarding whether there is a sex difference in TNF-α levels have been reported. In support of our data, Scheingraber *et al*. found no significant sex difference in TNF-α level of female and male patients undergoing major or minor abdominal surgery [20]. Similarly, Speyer *et al*. found no significant difference in TNF-α level between male and female mice after lipopolysaccharide-induced lung injury [21]. On the other hand, other studies have noted sex differences in TNF-α level [6,22]. The discrepancies between studies may be attributed to the experimental model applied, as well as species differences.

In addition to the cytokine TNF-α, the plasma IL-6 levels were also measured. Interleukin-6 is a multifunctional cytokine that is both pro-mitogenic and anti-apoptotic for hepatocytes, and is considered a marker for tissue injury severity [23,24]. In the current study, a significant difference in IL-6 levels between sham and hepatic I/R groups was observed (Figure 5). Initially female I/R mice had higher levels of IL-6 than males, which began to decline to lower levels than the males by 6 hrs of reperfusion (Figures 5A). Based on these observations it appears that females were primed to respond to hepatic injury, as initially they generated higher levels of IL-6. This priming condition may explain why female mice demonstrated less hepatic damage compared to the males (i.e. lower plasma ALT levels), and why females in general are somewhat more robust in response to sepsis and trauma than males [2]. This hypothesis has previously been suggested by Wichmann *et al*., who found an enhanced cytokine response in female mice after hemorrhage shock, compared to male mice [25]. Other studies have also shown a similar pattern [26]. Further, the initial increased IL-6 levels may be involved in anti-apoptotic actions and may add further support as to why females showed less hepatic damage. Teoh *et al*. reported that IL-6 may be a potential mediator of the hepatoprotective and pro-proliferative effects as a result of ischemic preconditioning [23]. Moreover, circulating estrogen may have lead to the decreased IL-6 levels, suggesting a potential explanation as to why IL-6 levels decreased in female I/R mice, while it continued to increase in male I/R mice [27].

The kinetics of chemokine production was examined in this study. Plasma MIP-2 levels in the sham group were constant and minimal, while a significant difference was observed between male and female hepatic I/R groups (Figure 6). Similar to the above noted cytokines, male I/R mice had significantly higher level of plasma MIP-2 than the female I/R counterparts. However, the most striking observation was the kinetic of KC production in the female mice. As Figure 7B shows, a significantly high level of plasma KC was present in the female sham mice at 1.5 hrs of reperfusion, which declined by 6 hrs of reperfusion. The plasma KC levels of the female sham group were significantly higher than those of the male counterparts at all time points of the reperfusion (Figure 7B). This response of the female sham group indicates an inflammatory response to the trauma induced by abdominal surgery (i.e., laparotomy), which was not noticeable in the male sham counterparts (Figure 7B). The heightened production of KC in females may be due to circulating estrogen, although the exact mechanisms by which estrogen exerts its effects are unknown. During the diestrous and metestrous stages, there is a marked increase in the number of leukocytes in the vaginal stroma and lumen, which denotes the presence of an inflammatory response. If the mice were at either of these stages of their estrous cycle, it may have primed KC production, and would account for the difference observed between male and female sham mice. To our knowledge, this is a novel observation that has not been previously reported, and further study is warranted to examine the regulation of the cytokine during the estrus cycle and its potential priming effect in inflammatory response. Specifically studies examining the different stages of the estrus cycle, as well as the use of ovariectomized female mice, the use of female mice administered an estrogen antagonist and the use of male mice administered estrogen will help elucidate estrogen's priming effect and its effect in hepatic IR injury. The data also suggested that under this experimental condition, CXC-chemokine production in female in response to laparotomy was not associated with TNF-α production since it's level was minute in the plasma of these sham mice. Further, the study suggested differential regulation of the two chemokines, i.e., KC, and MIP-2, in hepatic IR response. The current data regarding the plasma KC level in the male group is consistent with the previous reports indicating a constitutive expression of mRNA KC in the liver and presence of low levels of the protein in the plasma of male mice subjected to the sham operation [14,16]. The current study suggests that females may be primed to respond to injury, since there was a significantly greater increase in KC levels in females than the males. In addition, this study not only underscores the differential role of gender in cytokine regulation, but also highlights the importance of gender consideration in interpretations of the experimental data. The reader needs to be reminded that the data presented is preliminary and that further investigation is necessary in order to better elucidate the differential regulation of IL-6, MIP-2, and KC, as a result of gender dimorphism during hepatic I/R.

Although the exact mechanism to explain the differences in cytokine/chemokine levels after hepatic I/R injury is unknown, it may be postulated that the sex steroid hormones may play a role. In a study by Sener *et al*., pre-treatment with estradiol decreased levels of TNF-α in the liver and intestine of septic rats [3]. Estrogen inhibits TNF-α production both in vivo and in vitro [28-30]. Further, estrogen at physiological levels reduces IL-6, and MIP-2 production in many cell types, including macrophages [27,31,32]. Estrogen has also been shown to have an effect on endothelial nitric oxide (NO), in that it is able to increase production of NO [33]. An increase in NO would allow for vasodilatation to the site of hepatic injury and in turn facilitate reperfusion to the ischemic and surrounding tissue, minimizing the degree of tissue hypoxia and injury. Further, estrogen has strong antioxidant properties, including inhibition of low-density lipoprotein oxidation, decreased lipoprotein levels, and reduction in superoxide production [29,33,34]. Moreover, other studies have also shown that estrogen-receptor knock-out mice have significantly higher ALT levels than their wild-type female equivalents [17]. Collectively, these studies indicate a potential mechanism by which estrogen may reduce the extent of hepatic I/R injury. Further studies are required to establish the role of estrogen in hepatic IR by utilizing estrogen/estrogen receptor antagonists or knockout mice.

The present study is the first to demonstrate that under normal pathophysiologic condition there is a gender-specific response in cytokine/chemokine regulation as an inflammatory response to hepatic I/R. Based on the data presented here, it is evident that cytokine/chemokine regulation in an innate immune response to hepatic I/R displays a gender dimorphic response. The study also highlights the complex nature of I/R injury in which IL-6 and the chemokines MIP-2, and KC do not correlate with the hepatic injury in a cause-and-effect manner. The current findings warrant further investigation into the role of gender dimorphism and the underlying mechanisms of hepatic I/R injury during early and late phases of injury, and in addition, the potential therapeutic role of sex steroids in hepatic injury.

## Abbreviations

**ELISA**, enzyme linked immunosorbent assay; **IL**, interleukin; **IR**, ischemia/reperfusion; **MIP-2**, macrophage inflammatory protein-2; **ROS**, reactive oxygen species; **TNF-α**, tumor necrosis factor-α.

## Competing interests

The author(s) declare that they have no competing interests.

## Authors' contributions

EC was responsible for conceiving, designing and carrying out the project, as well as drafting the manuscript. WS and SD participated in the analysis and interpretation of the data, as well as drafting the manuscript. JH carried out the surgical procedures, collection of the tissue samples and ALT measurement. All authors read and approved the manuscript.
